# Phylogenetic relatedness determined between antibiotic resistance and 16S rRNA genes in actinobacteria

**DOI:** 10.1186/s12866-015-0416-6

**Published:** 2015-04-01

**Authors:** Marketa Sagova-Mareckova, Dana Ulanova, Petra Sanderova, Marek Omelka, Zdenek Kamenik, Jana Olsovska, Jan Kopecky

**Affiliations:** Epidemiology and Ecology of Microorganisms, Crop Research Institute, Prague, Czech Republic; Laboratory for Biology of Secondary Metabolism, Institute of Microbiology of the AS CR, v.v.i., Prague, Czech Republic; Faculty of Pharmacy, Charles University, Hradec Kralove, Czech Republic; Department of Probability and Mathematical Statistics, Faculty of Mathematics and Physics, Charles University, Prague, Czech Republic; Laboratory of Fungal Genetics and Metabolism, Institute of Microbiology of the AS CR, v.v.i., Prague, Czech Republic; Analytical and Testing Laboratory, Research Institute of Brewing and Malting, PLC, Prague, Czech Republic; Oceanography Section, Science Research Center, Kochi University, IMT-MEXT, Kochi, Japan

**Keywords:** Actinobacteria, 16S rRNA diversity, Resistance genes, Thin layer chromatography, Sequenced genome database, Phylogeny

## Abstract

**Background:**

Distribution and evolutionary history of resistance genes in environmental actinobacteria provide information on intensity of antibiosis and evolution of specific secondary metabolic pathways at a given site. To this day, actinobacteria producing biologically active compounds were isolated mostly from soil but only a limited range of soil environments were commonly sampled. Consequently, soil remains an unexplored environment in search for novel producers and related evolutionary questions.

**Results:**

Ninety actinobacteria strains isolated at contrasting soil sites were characterized phylogenetically by 16S rRNA gene, for presence of *erm* and ABC transporter resistance genes and antibiotic production. An analogous analysis was performed *in silico* with 246 and 31 strains from Integrated Microbial Genomes (JGI_IMG) database selected by the presence of ABC transporter genes and *erm* genes, respectively. In the isolates, distances of *erm* gene sequences were significantly correlated to phylogenetic distances based on 16S rRNA genes, while ABC transporter gene distances were not. The phylogenetic distance of isolates was significantly correlated to soil pH and organic matter content of isolation sites. In the analysis of JGI_IMG datasets the correlation between phylogeny of resistance genes and the strain phylogeny based on 16S rRNA genes or five housekeeping genes was observed for both the *erm* genes and ABC transporter genes in both actinobacteria and streptomycetes. However, in the analysis of sequences from genomes where both resistance genes occurred together the correlation was observed for both ABC transporter and *erm* genes in actinobacteria but in streptomycetes only in the *erm* gene.

**Conclusions:**

The type of *erm* resistance gene sequences was influenced by linkage to 16S rRNA gene sequences and site characteristics. The phylogeny of ABC transporter gene was correlated to 16S rRNA genes mainly above the genus level. The results support the concept of new specific secondary metabolite scaffolds occurring more likely in taxonomically distant producers but suggest that the antibiotic selection of gene pools is also influenced by site conditions.

**Electronic supplementary material:**

The online version of this article (doi:10.1186/s12866-015-0416-6) contains supplementary material, which is available to authorized users.

## Background

The lack of understanding the evolution and function of secondary metabolites in natural habitats constrains our abilities to develop strategies for antibiotic exploration, to manage indigenous soil microbial communities for enhanced inhibitory phenotypes or minimize accumulation of antibiotic resistance in both natural and human environments [1]. Yet, it has been recently demonstrated that individual types of secondary metabolites were correlated to the site of isolation [2,3] and to the producer’s phylogenetic origin [4]. For example, selection of a specific site led to a recent description of *Streptomyces* sp. NTK 937, producer of caboxamycin discovered in deep ocean [5] and with help of selective cultivation to *Streptomyces platensis*, producer of platensimycin, discovered in South African soil [6]. Those findings also indicated that even the most frequently isolated genus *Streptomyces* may still provide novel metabolites if less explored natural habitats are selected. The local specificity of *Streptomyces* was explained by horizontal acquisition of genes and selection of local phylogenetic lineages by local ecological context, thus creating a niche-specific gene pool [7].

The coexistence of production and resistance in individual organisms has been confirmed in both antibiotic biosynthetic gene clusters and examinations of the genome sequences from producing strains. With the exception of nonspecific efflux systems, the potential antibiotic resistance determinants found in antibiotic-producing strains were generally associated with antibiotic structural types or modes of action [8]. Indeed, selection for the specific producer-type resistance to enrich for producers of selected compounds and biosynthetic genes phylogeny to select for novel scaffolds was successfully applied to identify new producers of glycopeptide and ansamycin antibiotics [9]. Also, the selection of isolates based on the antibiotic mode of action was suggested as a preferred strategy compare to the original empirical screening for any detectable bioactivity [10].

The goal of this study was to characterize and compare actinobacterial strains isolated at natural soil sites in terms of their phylogenetic relationship, antibiotic production and possession of two selected resistance genes, *erm* and ABC transporter resistance genes. Those characteristics were also correlated to the environmental conditions of the isolation sites. Finally, it was suggested how to use this information in exploring the possibilities of novel antibiotic discovery.

The *erm* resistance genes code for 23S rRNA mono- and dimethyltransferases, and the ABC transporter genes code for ATPase subunit of type II-ABC transporters. The *erm* resistance represents a well characterized protein with known phylogeny [11] with a specific mode of action for an antibiotic targeting 23S rRNA. On the contrary, ABC transporters represent a more general efflux mechanism, in which the protein sequence corresponds rather with the structure of the transported antibiotic specified by its binding site. The two resistance types are of different phylogenetic and functional origin, and provide different information on the type of the corresponding antibiotic. However, they may occur in one biosynthetic cluster, as described for example in the production of lincomycin [12,13], tylosin [14,15] and spiramycin [16].

Isolation sites were selected by contrasting soil pH, organic matter content, bedrock and vegetation as the most important source of distinct carbon substrates [17]. Character of organic matter input was considered because streptomycetes change their antibiotic activities with respect to different carbon content and quality [18]. In particular, cellulose and lignin inputs increased population densities and number of antibiotic producer phenotypes suggesting enrichment of antibiotic-producing *Streptomyces* in nutrient-enriched habitats in soil [18]. Similar approach has been already described in several studies for characterization of less-explored or unexplored microorganisms with potentially useful biological activities [19]. The sites also differed in actinobacteria community composition, types and diversity of *erm* gene sequences [17] and percentage of bioactive isolates [20]. So, the selection of sampling sites complied with a suggestion that isolation of antibiotic producers should be based on understanding their ecological context to target the relevant microbial space [7].

In this work we evaluated the hypothesis that phylogenetic relationships of two different resistance genes will be linked with their different phylogenetic origin in actinobacteria and selection pressures of local conditions. The work aimed at exploring this hypothesis at three different levels including a random selection of strains isolated from soil sites covering a range of environmental factors, a set of characterized resistance genes known as parts of biosynthetic gene clusters, and more comprehensive, but functionally uncertain, set of homologous genes available from the sequenced genomes of actinobacteria.

## Methods

### Isolation of actinobacteria

Soil samples collected from 9 contrasting sites were used for isolation of actinomycetes. Sites and their characteristics are listed in Table 1. One gram of soil extracted with 10 ml of SET buffer, vortexed 2 min at 2500 rpm. Aliquots of 100 μl of each sample were plated on MPA medium (peptone, 5 g/l, beef extract, 3 g/l, agar, 15 g/l, pH 7.0) and R2A medium (yeast extract, 0.5 g/l, proteose peptone, 0.5 g/l, casamino acids, 0.5 g/l, glucose, 0.5 g/l, soluble starch, 0.5 g/l, Na-pyruvate, 0.3 g/l, K_2_HPO_4_, 0.3 g/l, MgSO_4_ · 7H_2_O, 0.05 g/l, agar, 15 g/l, pH adjusted to the original soil pH). Both media were also used ten times diluted and supplemented with soil extract (2× soil extract: 200 g of soil were boiled for 20 min in 250 ml of distilled H_2_O, filtered with a filter paper, cooled and adjusted to original soil pH). All agar plates contained cycloheximide (300 μg/ml) to prevent fungal growth. Plates were incubated for 12–24 days at 28°C. Strains forming colonies with aerial mycelium and/or submerged to the agar medium were considered putative actinobacteria, collected and purified by sequential streaking on two plates of the medium used for isolation with and without cycloheximide. 719 strains were collected. All isolates from the same site similar in morphology and bioactivity against *Escherichia coli* and *Kocuria rhizophila* were eliminated from this set of strains. The remaining 386 strains were tested for the presence of *erm* and ABC transporter genes with positive results in 1.8% (n = 7) and 4.7% (n = 18) of the tested strains, respectively. Ninety strains, which included all strains positive for either of the resistance genes and 67 other randomly selected strains were classified by 16S rRNA gene sequences and subjected to further analysis.

**Table 1:** **Site characteristics and numbers of isolated strains**

**Site**		**Soil pH**	**Soil organic matter [%]**	**Bedrock**	**Vegetation**		**Number of strains**	
					**Type**	**Dominant species**	**Total**	**Tested for resistance genes**
**Devin**	**DE**	7.9	12	limestone	grassland	*Poaceae*	45	42
**Merkenstein**	**ME**	8.1	16.5	dolomite	pine forest	*Pinus nigra*, forbs	135	114
**Nechranice**	**NE**	6.1	11.6	phonolite	deciduous forest	*Acer pseudoplatanus, Tilia cordata*	21	12
**Oblik**	**OB**	7.9	21.5	basalt	grassland	*Brassicaceae, Fabaceae, Asteraceae*	13	7
**Srbsko**	**SR**	7.7	8.2	limestone	deciduous forest	*Quercus robur, Carpinus betulus*	223	52
**Stampftal**	**ST**	7.9	14.8	dolomite	pine forest	*Pinus nigra, Poaceae*	81	62
**Trebon**	**TR**	4.0	9.1	granodiorite	deciduous forest	*Populus tremula*	171	73
**Zakopana**	**ZA**	3.7	3.1	sandstone	pine forest	*Pinus silvestris*	11	9

### Antibiotic activity test

Strains were inoculated on agar plates with sporulation GYM medium (glucose 4 g/l, yeast extract 4 g/l, malt extract 10 g/l, CaCO_3_ 2 g/l, pH 7.2, agar 15 g/l) and incubated for 10 days at 28°C. Agar plugs of 5 mm in diameter of each isolated strain were placed on B1 agar plates (beef extract 10 g/l, peptone 10 g/l, NaCl 5 g/l, agar 20 g/l, pH 7.2) with sensitive strains of *Kocuria rhizophila* CCM 552 (Czech Collection of Microorganisms, Czech Republic; equivalent to ATCC 9341) and *Escherichia coli* CCM 3988 (ATCC 10536). Plates were incubated overnight at 37°C and occurrence and size of inhibition zones were recorded.

### Cultivation and thin layer chromatography (TLC) analysis

Strains were inoculated from GYM plates to GYM liquid broth and grown on a rotary shaker for 24-48 h at 28°C, 250 rpm. Fresh GYM broth was inoculated with 5% of these cultures and cultivation continued for next 10 days at 28°C, 250 rpm. Cells were separated by centrifugation for 10 min at 10 000 × g, 4°C, and cultivation broth was used for TLC analyses. Thirty μl of cultivation broth were applied on silica plates (Merck, Germany) by Hamilton micro syringe and the plates were placed in a chromatographic chamber pre-saturated for minimum 1 h with a mobile phase consisting of chloroform : methanol : ammonia (28%), 12:3:0.1. Detection of low-molecular weight substances was performed using a UV lamp at 254 and 366 nm, and by staining with coloring reagent. For this purpose developed plates were dried for 30 min at room temperature prior to spraying by orcinol reagent (orcinol 250 mg, ethanol 44 ml and sulphuric acid 6 ml). The plates were subsequently heated at 110°C for 2 min and spot appearance, position, and color were determined. Retention factor (R_f_) values were determined by dividing distance moved by compound by distance moved by solvent.

### Primers

Degenerate primers rB1f, 5'-ARCWCGGYCAGAAYTTYCT-3', and rB1r, 5'-CGSGCSACYTCCCAYTG-3' [20] were used for amplification of *erm* gene. The primers designed to cover mainly the genes from GC rich Gram positive bacteria were validated in previous studies for amplification from complex environmental DNA samples [17,20]. *In silico* analysis of their coverage using OligoCheck software included in Bioinformatic toolkit [21] with the database of actinobacteria genomic sequences produced by the US Department of Energy Joint Genome Institute (http://www.jgi.doe.gov/), available at Integrated Microbial Genomes server (http://img.jgi.doe.gov/, further referenced as JGI_IMG database) confirmed their selectivity (Additional file 1: Figure S1a).

The degenerate primers rC4f, 5'-CTSGACGARCCSACYAA-3', and rC4r, 5'-GKYSGTSGGCTCGTC-3' (this work) for amplification of partial sequences of genes coding for ABC transporter ATPase subunit were designed using Primrose software included in Bioinformatic toolkit [21] based on known resistance determinants from biosynthetic clusters. The primers were validated by amplification from genomic DNA of selected collection strains and from complex environmental DNA samples (not shown). *In silico* analysis showed a broad coverage of actinobacteria ABC transporter resistance genes (Additional file 1: Figure S1b).

Primers for amplification of partial 16S rDNA were: 16Seu27f (5′AGAGTTTGATCMTGGCKCAG) [20] and 16Seu783r, an equimolar mix of 5′-CTACCAGGGTATCTAATCCTG-3', 5′-CTACCGGGGTATCTAATCCCG-3' and 5′-CTACCCGGGTATCTAATCCGG-3' [22].

### PCR

All PCR reactions were performed with a TGRADIENT Thermocycler (Whatman Biometra, Germany). Modified protocol for colony PCR amplification was used [23] with mycelium from GYM medium as a template. Reaction mixture contained in a total volume of 50 μl: 1× polymerase buffer, 1.5 mM MgCl_2_, 400nM of each primer, 0.2 mM of each dNTP, 0.6 mg/ml BSA, 5% DMSO, 1% Nonidet P40 (Sigma-Aldrich, MO, USA), 2.5U LA polymerase (TopBio, Czech Republic). PCR program consisted of an initial denaturation at 94°C for 5 min, followed by 35 cycles of 94°C for 1 min, 56°C for 45 s, 68°C for 1.5 min and final extension for 5 min at 68°C. The PCR products were purified and sequenced by Macrogen (Korea).

### Datasets

Sequences of *erm* and ABC transporter genes included in the analysis were selected based on (i) published data on the *erm* [13-16,24-32] and ABC transporter [33] genes sequenced either as parts of antibiotic biosynthetic gene clusters or as independent resistance determinants and deposited in GenBank, or (ii) BLAST search in the database of 306 complete or draft genomes of actinobacteria from the JGI_IMG database. The homologous proteins were searched for using blastp algorithm and *Streptomyces lincolnensis erm*-class 23S rRNA methyltransferase LmrB [GenBank: ABX00620] and ATPase subunit of type II-ABC transporter LmrC [GenBank: ABX00624] as the query sequences.

**Figure 1:**
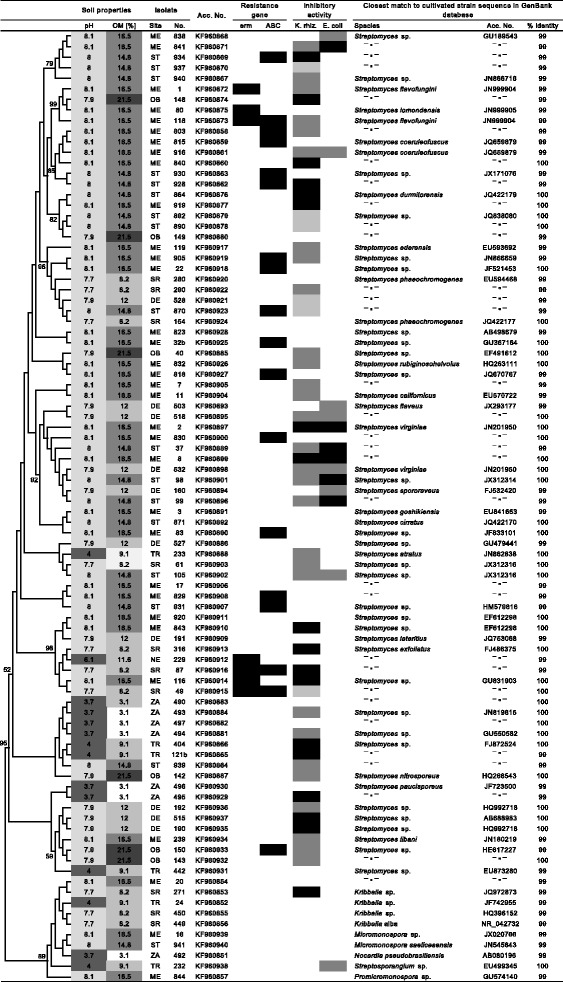
**Isolated strains with their phylogeny, origin, occurrence of the resistance genes and inhibitory activity against** ***K. rhizophilla*** **and** ***E. coli.*** The isolation sites are depicted by abbreviations as in Table 1. The phylogram was constructed by neighbor-joining method based on Jukes-Cantor distance matrix calculated from alignment of 16S rRNA gene sequences. Branch lengths correspond to the evolutionary distances. Branch labels indicate the supporting bootstrap values based on analysis of 1000 replicates. The inhibitory activity is marked by shades of grey, from light to dark: inhibitory zones below 10 mm, 10-15 mm, and above 15 mm in diameter.

The search for Erm protein homologs retrieved putative Erm proteins with the highest degree of homology (35% identities or higher), closely related KsgA proteins (up to 30% identities) and several groups of unspecified methyltransferases, which were excluded based on low similarity. The KsgA proteins could be precisely identified and excluded by comparison of the genomic context of the encoding genes, which was highly conserved throughout actinobacteria, with neighboring genes coding for 4-diphosphocytidyl-2-C-methyl-D-erythritol kinase, putative deoxyribonuclease related to TatD family hydrolases, and methionyl-tRNA synthetase. The selection yielded a set 31 defined or putative Erm proteins from actinobacterial strains with available 16S rRNA gene sequence (Figure 1, Additional file 1: Figure S2). In those genomes, ABC transporter gene fulfilling the selection conditions was always present, so the dataset 31 is a subset of the dataset 246.

The search for ABC transporter type II ATPase subunits related to LmrC retrieved several matching gene products with high homology in most of the genomes. For the analysis, only the closest LmrC homolog with at least the threshold score of 40% positives was selected from each strain, resulting in a set of 246 ABC transporter ATPase subunit proteins from actinobacterial strains with available 16S rRNA gene sequence (Additional file 1: Figure S3).

### Data analysis

Alignments of partial 16S rRNA, *erm*, ABC transporter, and housekeeping genes sequences were performed using Muscle 3.6 [34]. For large sequence datasets, phylogeny was inferred by neighbor-joining method based on Jukes-Cantor (for nucleotide sequences) or Jones-Taylor-Thornton (for amino-acid sequences) distance matrices calculated for 1000 bootstrap resamplings in Phylip 3.69 package [35]. For precise analysis of phylogenetic relationships of resistance genes sequenced from the isolates, the best-fit models of protein sequence evolution were selected using ProtTest v.3.2 [36]. Based on the selection, models WAG [37] with a proportion of invariable sites (+I), gamma distribution with four rate categories (+G), and observed amino acid frequencies (+F) and LG + G + F [38] were employed in analysis of Erm and ABC transporter amino-acid sequences, respectively. In that case, phylogenies were inferred by Bayesian analysis using PhyloBayes-MPI, v.1.4e [39] and maximum-likelihood analysis in PhyML v.3.0 [40].

All the statistical computations were conducted within the R statistical computing environment (http://www.r-project.org). Distance matrices of resistance genes, 16S rRNA genes, and housekeeping genes sequences were calculated with Phylip 3.69 software [35]. Correlation analysis was based on generalized distance covariance test [41] based on the method developed by Szekely et al. [42]. As the aim of the study is explanatory, no correction of p-values for multiple testing was used. It is, therefore, necessary to bear in mind that the p-values below or equal to 0.001 provide strong evidence, while the p-values between 0.001 and 0.05 need to be confirmed by an independent study.

### Data deposition

The sequences obtained in this study were deposited in GenBank under accession numbers KF960851 - KF960940 for 16S rRNA genes, KF960941 - KF960947 for *erm* genes, and KF960948 - KF960964 for ABC transporter genes.

The data of the phylogenetic analyses are available from the Dryad Digital Repository: http://dx.doi.org/10.5061/dryad.td742.

## Results

### Isolates

**Figure 2:**
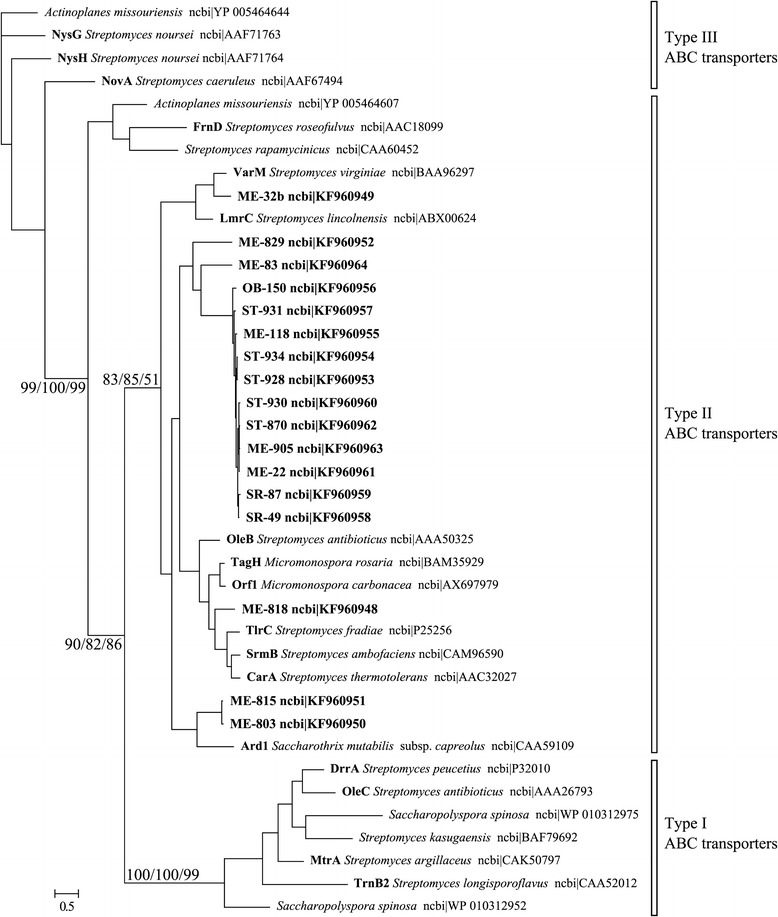
**Phylogenetic analysis of 17 putative ABC transporters from the isolated strains with 23 reference sequences of ABC transporters described in actinobacteria.** The phylogeny was inferred by Bayesian analysis of amino-acid sequence alignment. Branch lengths correspond to mean posterior estimates of evolutionary distances (scale bar, 0.5). Branch labels indicate the Bayesian posterior probability and supporting bootstrap values from maximum-likelihood and neighbor-joining analyses for branches with significant support and relevance for clustering of the analyzed sequences.

Out of the 90 selected isolates, 16S rRNA gene sequences of 37 strains showed 100% similarity, and 53 strains 99% similarity to the closest isolated strain match in the GenBank database (Figure 2). The strains were assigned to the genus *Streptomyces* (79 isolates) and to the genera *Kribella*, *Hongia*, *Micromonospora*, *Nocardioides*, *Nocardia*, *Promicromonospora* and *Streptosporangium*. The phylogenetic relatedness of the isolates correlated with soil properties at the sampling sites (Figure 2), namely with soil pH (p = 0.004) and organic matter content (p = 0.001).

### *Erm* and ABC transporter genes

**Figure 3:**
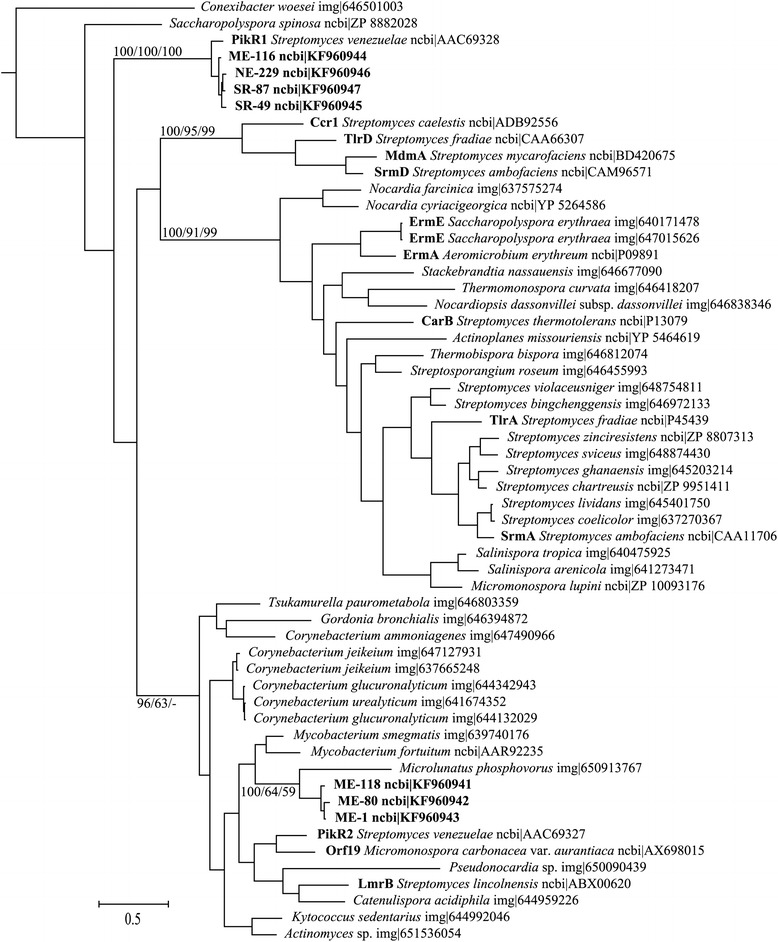
**Phylogenetic analysis of 7 putative** ***erm*** **genes from the isolated strains obtained in this study with 50 actinobacterial described or putative** ***erm*** **genes.** The phylogeny was inferred by Bayesian analysis of amino-acid sequence alignment. Branch lengths correspond to mean posterior estimates of evolutionary distances (scale bar, 0.5). Branch labels indicate the Bayesian posterior probability and supporting bootstrap values from maximum-likelihood and neighbor-joining analyses for branches with significant support and relevance for clustering of the analyzed sequences. The phylograms were outgrouped using KsgA sequence [Genbank: WP_003915138].

**Figure 4:**
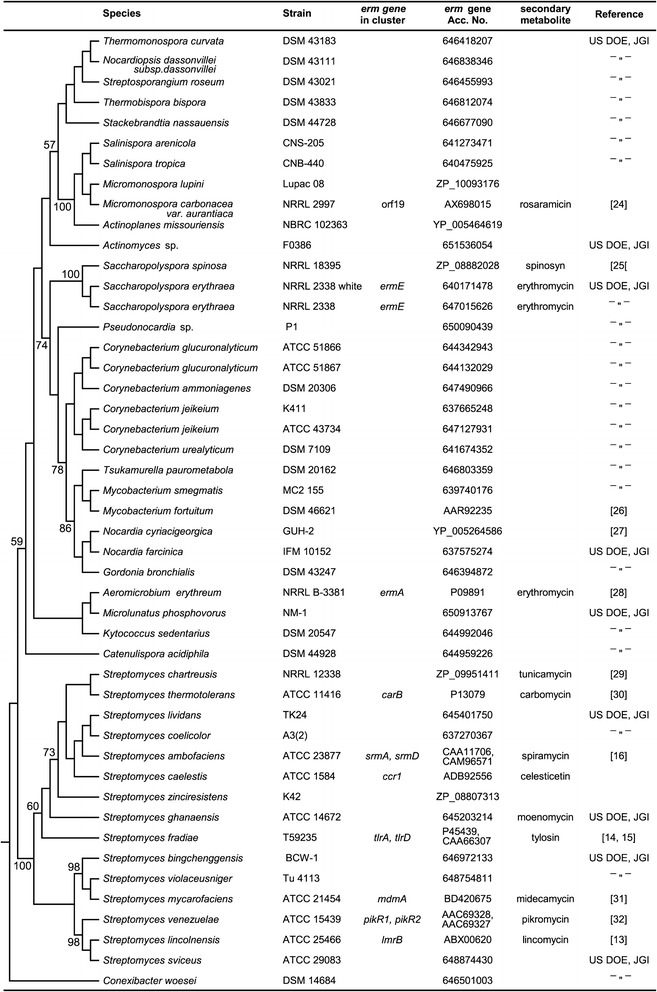
**Phylogenetic relationships of 47 collection strains harboring** ***erm*** **resistance genes.** Strains used in the analysis were selected from GenBank and JGI_IMG databases based on availability of both *erm* and 16S rRNA gene sequences. The phylogram was constructed by neighbor-joining method based on Jukes-Cantor distance matrix calculated from alignment of 16S rRNA gene sequences. Branch labels indicate the supporting bootstrap values based on analysis of 1000 replicates.

Sequences of *erm* and ABC transporter genes found in the isolates clustered together with previously described genes of the known function (Figures 3, 4). Phylogenetic distances of *erm* genes from streptomycete species were significantly related to phylogenetic distances based on the 16S rRNA gene sequences from the same strains (p = 0.005), while for ABC transporter genes, a similar correlation was not observed (Table 2). The two genes occurred more often together than separately (p = 0.049). The *erm* sequences formed two separate clusters. Three sequences of the first cluster came from ME site; while the second cluster was formed by one sequence from ME site, two from SR site, and one from NE site. Six out of seven strains positive for *erm* genes and all 17 strains positive for ABC transporter genes were isolated at sites with high pH limestone-dolomite bedrock soil (Figure 2).

**Table 2:** **Correlations between distance matrices of resistance gene and 16S rRNA gene sequences**

**Dataset**	**Correlated sequences**	**Taxonomic groups**	**Number of strains**	**R**	**p**
isolates	ABC transporter × 16S rRNA	streptomycetes	17	0.61	0.072
	*erm* × 16S rRNA	streptomycetes	7	1.00	0.005
JGI_IMG	ABC transporter × 16S rRNA	actinobacteria	246	0.66	< 0.001
		streptomycetes	16	0.79	0.001
JGI_IMG	*erm* × 16S rRNA	actinobacteria	31	0.82	< 0.001
		streptomycetes	6	0.96	0.007
JGI_IMG	ABC transporter × 16S rRNA	actinobacteria	31	0.76	< 0.001
(subset with *erm*)		streptomycetes	6	0.82	0.094
GB (in cluster)	ABC transporter × 16S rRNA	actinobacteria	6	0.76	0.333
	*erm* × 16S rRNA	actinobacteria	11	0.82	0.111
		streptomycetes	7	0.83	0.800

### Database analysis

All sequences in our datasets selected from the JGI_IMG database clustered together with resistance genes sequences with proved function in phylogenetic analysis (Additional file 1: Figures S2, S3). A significant correlation was found between ABC transporter genes and respective 16S rRNA genes in both actinobacteria (p < 0.001) and streptomycetes (p = 0.001) in genomes of JGI_IMG (246). In a subset of 31 JGI_IMG genomes, in which *erm* and ABC transporter genes occur together, ABC transporter genes were significantly correlated with the respective 16S rRNA gene sequences only for actinobacteria (p < 0.001) but not for streptomycetes, while *erm* genes were correlated with 16S rRNA for both actinobacteria (p < 0.001) and streptomycetes (p = 0.007). Sequences of both *erm* and ABC transporter genes were correlated to each other for actinobacteria (p < 0.001) and streptomycetes (p = 0.016). In contrast, in the dataset constructed of GenBank sequences from proved antibiotic biosynthetic pathways ABC transporter or *erm* genes were not correlated with the respective 16S rRNA genes for either actinobacteria or streptomycetes (Table 2, Figure 1).

From the 246 genomes of JGI_IMG, a subset of 238 genomes with available sequences of the genes *rpoB*, *recA*, *atpD*, *dnaA*, and *ftsZ* was selected. The amino-acid sequences of the five genes were used to calculate multi-locus sequence (MLS) phylogenetic distances. The resistance genes distances were compared to the MLS distances using a similar scheme as for the 16S rRNA gene (Table 3). A significant correlation was found for ABC transporter genes in both actinobacteria (p < 0.001) and streptomycetes (p = 0.006) in the entire set of 238 genomes. In a subset of 31 JGI_IMG genomes, in which *erm* and ABC transporter genes occur together, a significant correlation was observed for ABC transporter genes were in actinobacteria (p < 0.001) but not in streptomycetes, while *erm* genes were correlated for both actinobacteria (p < 0.001) and streptomycetes (p = 0.008) (Table 3).

**Table 3:** **Correlations between distance matrices of resistance gene and concatenated sequences of five housekeeping genes (MLS) in the JGI_IMG dataset**

**Dataset**	**Correlated sequences**	**Taxonomic groups**	**Number of strains**	**R**	**p**
JGI_IMG	ABC transporter × MLS	actinobacteria	238	0.65	< 0.001
		streptomycetes	13	0.81	0.006
JGI_IMG	*erm* × MLS	actinobacteria	31	0.81	< 0.001
		streptomycetes	6	0.97	0.008
JGI_IMG	ABC transporter × MLS	actinobacteria	31	0.76	< 0.001
(subset with *erm*)		streptomycetes	6	0.76	0.320

### Antibiotic production

Out of the 90 selected isolates, 44 produced compounds with activity against *K. rhizophila*, 3 against *E. coli*, 10 against both microorganisms and 33 were non-producing strains (Figure 2). The percentage of producing strains differed between sites; alkaline sites had higher proportion of bioactive isolates than acidic sites (Additional file 1: Figure S4). Thin layer chromatography analysis of cultivation media showed a significantly higher number of detected low-molecular-weight compounds in isolates coming from acidic compare to alkaline soils (p = 0.007, Additional file 1: Figure S5). Fisher's exact test showed that production of antibiotics active against *E. coli* was negatively associated to the presence of ABC transporter genes (p = 0.034).

## Discussion

Several different mechanisms contributed to the relationships between the phylogenies of two selected resistance genes and 16S rRNA genes in actinobacteria. In defined biosynthetic clusters (common for both genes), no correlation between both resistance gene sequences and sequences of 16S rRNA genes was found. This finding conforms to the mechanism of horizontal transfer expected in secondary metabolite pathways [43,44].

In our isolates and the dataset, where the two genes occurred together in one genome, *erm* genes but not ABC transporter genes were related to the respective 16S rRNA genes and the difference in phylogeny of the two genes occurred only in streptomycetes not actinobacteria. So, the two resistance genes showed different phylogenetic relationships, *erm* gene being more related to the phylogenetic origin of the respective strain. The difference in ABC transporter gene phylogeny observed in streptomycetes may be explained by increased horizontal transfer proposed within *Streptomyces* genus [45]. That agrees with the previously proposed model, in which recombination is acting as a cohesive force, which diminishes with increasing sequence divergence [46].

For *erm* gene, the observed relationship suggests that individual forms of the resistance genes evolved in separate lineages. This phenomenon was documented for streptomycin and viomycin producing streptomycetes where the occurrence of production and resistance correlated with phylogenetic relatedness based on multilocus sequence analysis [4]. Another explanation might be that the horizontal gene exchange is rapid in ecologically cohesive populations and maintains close relationship between community members [7,47,48]. Further to that, in our isolates 16S rRNA gene sequences were significantly correlated to soil pH and organic matter. That corresponded to our previous finding by molecular approaches at four of the isolation sites, where T-RFLP of 16S rRNA was strongly correlated to soil pH and organic matter and also to T-RFLP of *erm* genes [17]. This leads to a model of ecological speciation via genetic isolation triggered by habitat selection of nascent populations proposed by Polz *et al.* [7].

These findings may be influenced by some methodological limitations. Firstly, high degree of 16S rRNA gene homology may cause that the distances between 16S rRNA genes cannot correctly describe the phylogenetic relationships within lower taxa. However, the impact of this constrain did not seem relevant for the present study as the correlation of phylogenetic distances of resistance genes to the concatenation of five housekeeping genes gave the same results as the that to the 16S rRNA gene in JGI_IMG dataset. Secondly, the precision of sequence selection to the JGI_IMG datasets differed between the two resistance genes. The *erm* genes were selected by their similarity to *lmrB* gene and by genomic context, *i.e.* the conserved neighboring genes were used to exclude the closely related *ksgA* genes. A similar approach could not be applied to ABC transporter genes because several homologous genes of unknown function were present in the majority of genomes and they occurred in varying genomic contexts. Consequently, the ABC transporter genes were selected based on homology to *lmrC* gene sequence, and only one gene of the highest similarity in each genome was selected for the analysis. This was done to approach the true antibiotic resistance genes, i.e. those often shared horizontally.

All sequences of our datasets, which were selected from the JGI_IMG database clustered together with resistance genes sequences with proved function (Additional file 1: Figures S2, S3). Since no evidence for transfer of antibiotic resistance genes from the antibiotic producing strains to pathogenic or commensal bacteria was found [11] we assumed that the majority of resistance genes selected for our datasets originated in antibiotic producers. This enables a partial linking between our results based on genotypes and phenotypes. Phenotypes of our isolates were similarly dependent on environmental factors because higher proportion of bioactive strains was determined in alkaline soils similarly as in other studies [49]. However, higher number of detected compounds was found in acidic soils and even though this result is based only on a small dataset, we think that acidic soils may harbor novel actinobacterial diversity. That result is supported by our previous description of high *erm* gene variability and of a new suborder of actinobacteria dominating actinobacterial communities at both of the studied acidic sites (Trebon - [20,50]; Zakopana - [17]).

In our study it was shown that site selection is closely related to phylogenetic relationships of both 16S rRNA and resistance genes even within streptomycetes. Since our isolates belonged mostly to the *Streptomyces* genus their characterization parallels the debate on taxonomic diversity as a surrogate for chemical diversity [51].

## Conclusions

In conclusion, most of the industrially used strains have been isolated by classical cultivation methods from neutral or slightly alkaline soils and strains of acidophilic actinomycetes were rarely described [52]. In our isolates, the phylogenetic origin of strains strongly depended on soil properties such as pH and organic matter content suggesting that isolation from specific sites may yield taxonomically distinguished strains even at sub-genus level. Considering our results of resistance genes correlation with actinobacteria taxonomy and site characteristics together with previously reported relationships between secondary metabolic gene pool and both strain phylogeny [4] and site specificity [47,17,20], isolates from specific, particularly acidic soil sites may still represent a reservoir of unknown antibiotics and other secondary metabolites.

## Additional file

Additional file 1:
**Figure S1.** *In silico* analysis of primer coverage with sequence sets retrieved from 306 genomes of *Actinobacteria* in JGI_IMG database. **Figure S2.** Phylogenetic analysis of actinobacterial methyltransferase sequences related to Erm. **Figure S3.** Phylogenetic analysis of actinobacterial ABC transporter sequences. **Figure S4.** The proportion of strains expressing antibiotic activity. **Figure S5.** Low molecular weight compounds detected by TLC analysis.

## References

[1] Jousset A, Eisenhauer N, Materne E, Scheu S (2013). Evolutionary history predicts the stability of cooperation in microbial communities. Nature Comm.

[2] Ziemert N, Lechner A, Wietz M, Millán-Aguiñaga N, Chavarria KL, Jensen PR (2014). Diversity and evolution of secondary metabolism in the marine actinomycete genus *Salinispora*. Proc Natl Acad Sci U S A.

[3] Reddy BV, Kallifidas D, Kim JH, Charlop Powers Z, Feng Z, Brady SF (2012). Natural product biosynthetic gene diversity in geographically distinct soil microbiomes. Appl Environ Microbiol.

[4] Laskaris P, Tolba S, Calvo-Bado L, Wellington L (2010). Coevolution of antibiotic production and counter-resistance in soil bacteria. Environ Microbiol.

[5] Hohmann C, Schneider K, Bruntner C, Irran E, Nicholson G, Bull AT (2009). Caboxamycin, a new antibiotic of the benzoxazole family produced by the deep-sea strain *Streptomyces* sp. NTK 937. J Antibiot.

[6] Genilloud O, Gonzalez I, Salazar O, Martin J, Tormo JR, Vicente F (2011). Current approaches to exploit actinomycetes as a source of novel natural products. J Ind Microbiol Biotechnol.

[7] Polz MF, Alm EJ, Hanage WP (2013). Horizontal gene transfer and the evolution of bacterial and archaeal population structure. Trends Genet.

[8] Davies J, Davies D (2010). Origins and evolution of antibiotic resistance. Microbiol Mol Biol Rev.

[9] Thaker MN, Wang W, Spanogiannopoulos P, Waglechner N, King AM, Medina R (2013). Identifying producers of antibacterial compounds by screening for antibiotic resistance. Nat Biotechnol.

[10] Chopra I, Hesse L, O'Neill AJ (2002). Exploiting current understanding of antibiotic action for discovery of new drugs. J Appl Microbiol.

[11] Aminov RI, Mackie RI (2007). Evolution and ecology of antibiotic resistance genes. FEMS Microbiol Lett.

[12] Peschke U, Schmidt H, Zhang H-Z, Piepersberg W (1995). Molecular characterization of the lincomycin-production gene cluster of *Streptomyces lincolnensis* 78–11. Mol Microbiol.

[13] Koberska M, Kopecky J, Olsovska J, Jelinkova M, Ulanova D, Man P (2008). Sequence analysis and heterologous expression of the lincomycin biosynthetic cluster of the type *strain Streptomyces lincolnensis* ATCC 25466. Folia Microbiol.

[14] Kamimiya S, Weisblum B (1988). Translational attenuation control of *ermSF*, an inducible resistance determinant encoding rRNA N-methyltransferase from *Streptomyces fradiae*. J Bacteriol.

[15] Gandecha AR, Cundliffe E (1996). Molecular analysis of *tlrD*, an MLS resistance determinant from the tylosin producer, *Streptomyces fradiae*. Gene.

[16] Pernodet JL, Gourmelen A, Blondelet-Rouault MH, Cundliffe E (1999). Dispensable ribosomal resistance to spiramycin conferred by *srmA* in the spiramycin producer *Streptomyces ambofaciens*. Microbiology (Reading, Engl).

[17] Sagova-Mareckova M, Omelka M, Cermak L, Kamenik Z, Olsovska J, Hackl E (2011). Microbial communities show parallels at sites with distinct litter and soil characteristics. Appl Environ Microbiol.

[18] Schlatter D, Fubuh A, Xiao K, Hernandez D, Kinkel L (2009). Resource amendments influence density and competitive phenotypes of streptomyces in soil. Microb Ecol.

[19] Bredholdt H, Galatenko OA, Engelhardt K, Fjaervik E, Terekhova LP, Zotchev SB (2007). Rare actinomycete bacteria from the shallow water sediments of the Trondheim fjord, Norway: isolation, diversity and biological activity. Environ Microbiol.

[20] Cermak L, Kopecky J, Novotna J, Omelka M, Parkhomenko N, Plhackova K (2008). Bacterial communities of two contrasting soils reacted differently to lincomycin treatment. Appl Soil Ecol.

[21] Ashelford KE, Weightman AJ, Fry JC (2002). PRIMROSE: a computer program for generating and estimating the phylogenetic range of 16S rRNA oligonucleotide probes and primers in conjunction with the RDP-II database. Nucleic Acids Res.

[22] Sakai M, Matsuka A, Komura T, Kanazawa S (2004). Application of a new PCR primer for terminal restriction fragment length polymorphism analysis of the bacterial communities in plant roots. J Microbiol Methods.

[23] Gathogo EWN, Waugh ACW, Peric N, Redpath MB, Long PF (2003). Colony PCR amplification of actinomycete DNA. J Antibiot.

[24] Farnet CM, Staffa A, Yang X. Genes and proteins for the biosynthesis of rosaramicin. Patent 2003; WO 03010193-A2.

[25] Pan Y, Yang X, Li J, Zhang R, Hu Y, Zhou Y (2011). Genome sequence of the spinosyns-producing bacterium *Saccharopolyspora spinosa* NRRL 18395. J Bacteriol.

[26] Nash KA, Zhang Y, Brown-Elliott BA, Wallace RJ (2005). Molecular basis of intrinsic macrolide resistance in clinical isolates of *Mycobacterium fortuitum*. J Antimicrob Chemother.

[27] Zoropogui A, Pujic P, Normand P, Barbe V, Beaman B, Beaman L (2012). Genome sequence of the human- and animal-pathogenic strain *Nocardia cyriacigeorgica* GUH-2. J Bacteriol.

[28] Roberts AN, Hudson GS, Brenner S (1985). An erythromycin-resistance gene from an erythromycin-producing strain of *Arthrobacter* sp. Gene.

[29] Doroghazi JR, Ju KS, Brown DW, Labeda DP, Deng Z, Metcalf WW (2011). Genome sequences of three tunicamycin-producing *Streptomyces* strains, *S. chartreusis* NRRL 12338, *S. chartreusis* NRRL 3882, and *S. lysosuperificus* ATCC 31396. J Bacteriol.

[30] Epp JK, Burgett SG, Schoner BE (1987). Cloning and nucleotide sequence of a carbomycin-resistance gene from *Streptomyces thermotolerans*. Gene.

[31] Mido N, Hoshigo S, Murakami T. Midecamycin biosynthetic gene cluster. Patent 2004; JP 2004049100-A1.

[32] Xue Y, Zhao L, Liu HW, Sherman DH (1998). A gene cluster for macrolide antibiotic biosynthesis in *Streptomyces venezuelae*: architecture of metabolic diversity. Proc Natl Acad Sci U S A.

[33] Mendez C, Salas JA (2001). The role of ABC transporters in antibiotic-producing organisms: drug secretion and resistance mechanisms. Res Microbiol.

[34] Edgar RC (2004). MUSCLE: multiple sequence alignment with high accuracy and high throughput. Nucleic Acids Res.

[35] Felsenstein J (1989). PHYLIP-Phylogeny inference package (version 3.2). Cladistics.

[36] Darriba D, Taboada GL, Doallo R, Posada D (2011). ProtTest 3: fast selection of best-fit models of protein evolution. Bioinformatics.

[37] Whelan S, Goldman N (2001). A general empirical model of protein evolution derived from multiple protein families using a maximum-likelihood approach. Mol Biol Evol.

[38] Le SQ, Gascuel O (2008). An improved general amino acid replacement matrix. Mol Biol Evol.

[39] Lartillot N, Lepage T, Blanquart S (2009). PhyloBayes 3: a Bayesian software package for phylogenetic reconstruction and molecular dating. Bioinformatics.

[40] Guindon S, Dufayard JF, Lefort V, Anisimova M, Hordijk W, Gascuel O (2010). New Algorithms and Methods to Estimate Maximum-Likelihood Phylogenies: Assessing the Performance of PhyML 3.0. Syst Biol.

[41] Omelka M, Hudecova S (2013). A comparison of the Mantel test with a generalised distance covariance test. Environmetrics.

[42] Szekely GJ, Rizzo ML, Bakirov NK (2007). Measuring and testing independence by correlation of distances. Ann Stat.

[43] Baltz RH, Miao V, Wrigley SK (2005). Natural products to drugs: daptomycin and related lipopeptide antibiotics. Nat Prod Rep.

[44] Egan S, Wiener P, Kallifidas D, Wellington EMH (2001). Phylogeny of *Streptomyces* species and evidence for horizontal transfer of entire and partial antibiotic gene clusters. Antonie Van Leeuwenhoek.

[45] Doroghazi JR, Buckley DH (2010). Widespread homologous recombination within and between *Streptomyces* species. ISME J.

[46] Fraser C, Hanage WP, Spratt BG (2007). Recombination and the nature of bacterial speciation. Science.

[47] Davelos AL, Kinkel LL, Samac DA (2004). Spatial variation in frequency and intensity of antibiotic interactions among streptomycetes from prairie soil. Appl Environ Microbiol.

[48] Fischbach MA, Walsh CT, Clardy J (2008). The evolution of gene collectives: How natural selection drives chemical innovation. Proc Natl Acad Sci U S A.

[49] Gonzalez I, Niebla A, Lemus M, Gonzalez L, Iznaga IO, Perez ME (1999). Ecological approach of macrolide-lincosamides-streptogramin producing Actinomyces from Cuban soils. Lett Appl Microbiol.

[50] Kopecky J, Kyselkova M, Omelka M, Cermak L, Novotna J, Grundmann GL (2011). Actinobacterial community dominated by a distinct clade in acidic soil of a waterlogged deciduous forest. FEMS Microbiol Ecol.

[51] Jensen PR (2010). Linking species concepts to natural product discovery in the post-genomic era. J Ind Microbiol Biotechnol.

[52] Zakalyukina YV, Zenova GM (2007). Antagonistic activity of soil acidophilic actinomycetes. Biol Bull.

