# Supplementary material for: Phylogenetic relatedness determined between antibiotic resistance and 16S rRNA genes in actinobacteria
Source: BMC Microbiol. 2015 Apr 1;15:81. doi: 10.1186/s12866-015-0416-6 (PMC4391685; doi:10.1186/s12866-015-0416-6)

Sagova-Mareckova et al.
Phylogenetic relatedness determined between antibiotic resistance and 16S rRNA genes in actinobacteria.

Additional file

Figure S1. ***In silico*** **analysis of primer coverage with sequence sets retrieved from 306 genomes of** ***Actinobacteria*** **in JGI_IMG database.**

(a) Primers rC4f and rC4r against a database of 1097 putative ABC transporters.
(b) Primers rB1f and rB1r against a database of 31 erm genes (target group, blue circles) and 470 sequences of other putative methyltransferases (outgroup, red triangles).

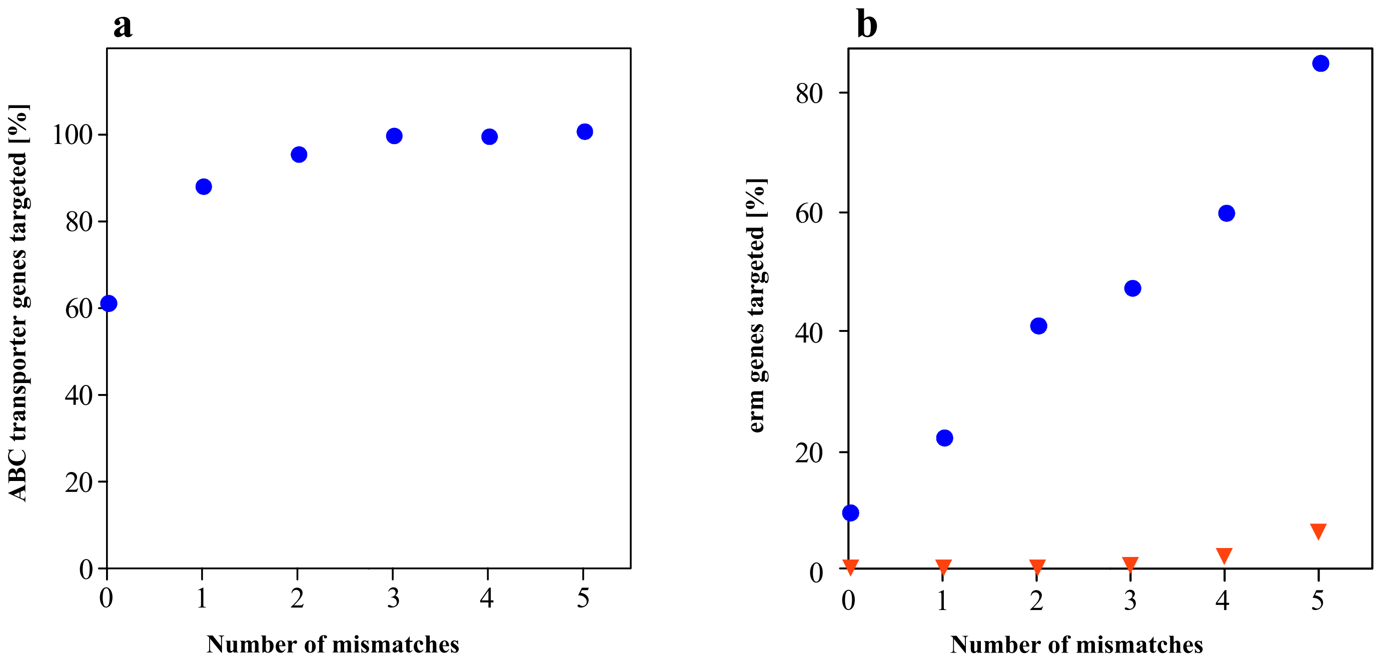

Figure S2. Phylogenetic analysis of actinobacterial methyltransferase sequences related to Erm.

Phylogeny was inferred by Neighbor-Joining method based on Jones-Taylor-Thornton distance matrix calculated from initial alignment of 510 full-length amino-acid sequences. Among the selected sequences, 19 were described Erm resistance proteins retrieved from GenBank database, and 491 were retrieved from JGI-IMG database based on homology. Branch labels indicate the percentage of resampling replicates supporting the respective branch.

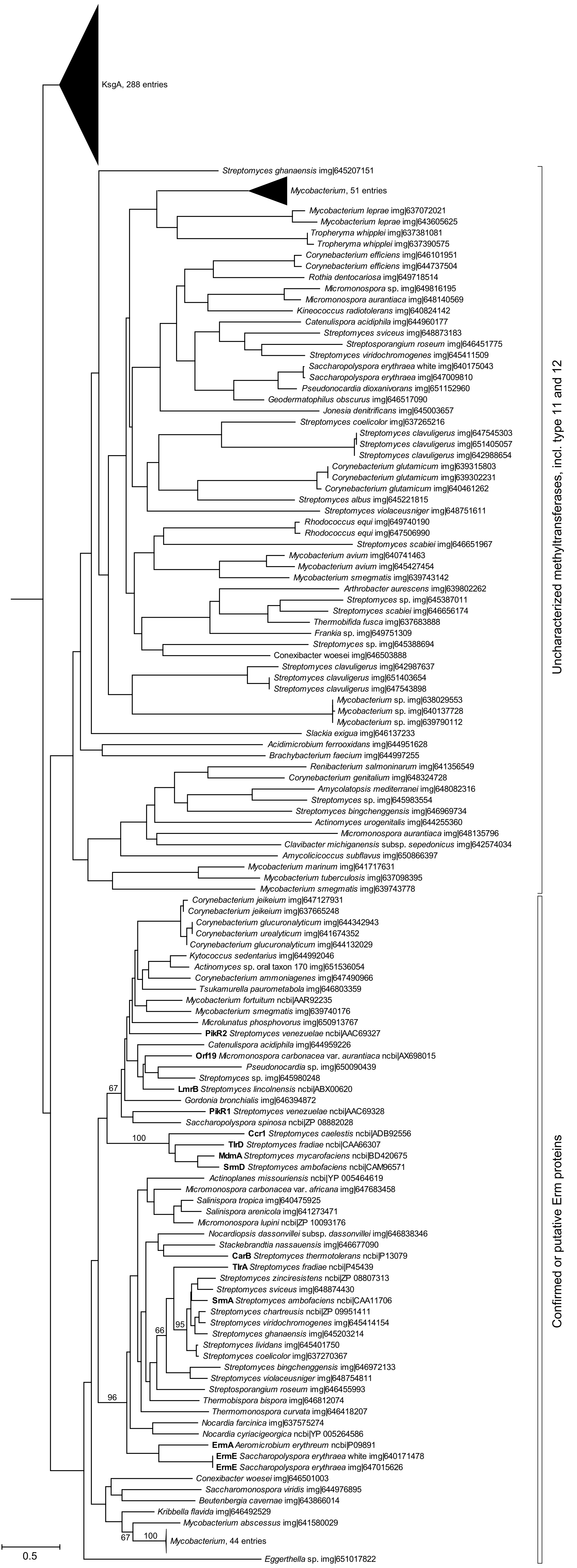

Figure S3. Phylogenetic analysis of actinobacterial ABC transporter sequences.

Phylogeny was inferred by Neighbor-Joining method based on Jones-Taylor-Thornton distance matrix calculated from initial alignment of 274 full-length amino-acid sequences. Among the selected sequences, 28 were known ABC transporter resistance genes, and 246 were retrieved from JGI-IMG database based on homology. Branch labels indicate the percentage of resampling replicates supporting the respective branch.

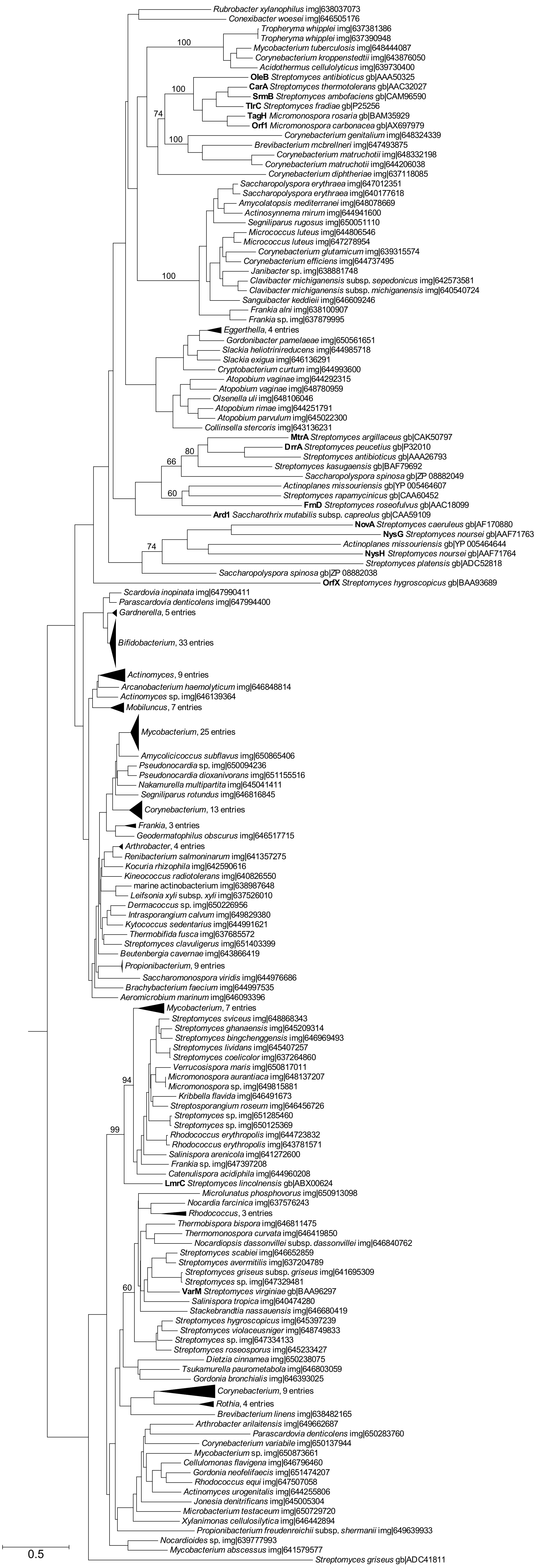

Figure S4. The proportion of strains expressing antibiotic activity.

The columns indicate a percentage of strains inhibiting *Kocuria rhizophilla* (black) and *Escherichia coli* (empty) among isolates from individual alkaline (blue) and acidic (red) soil sites.

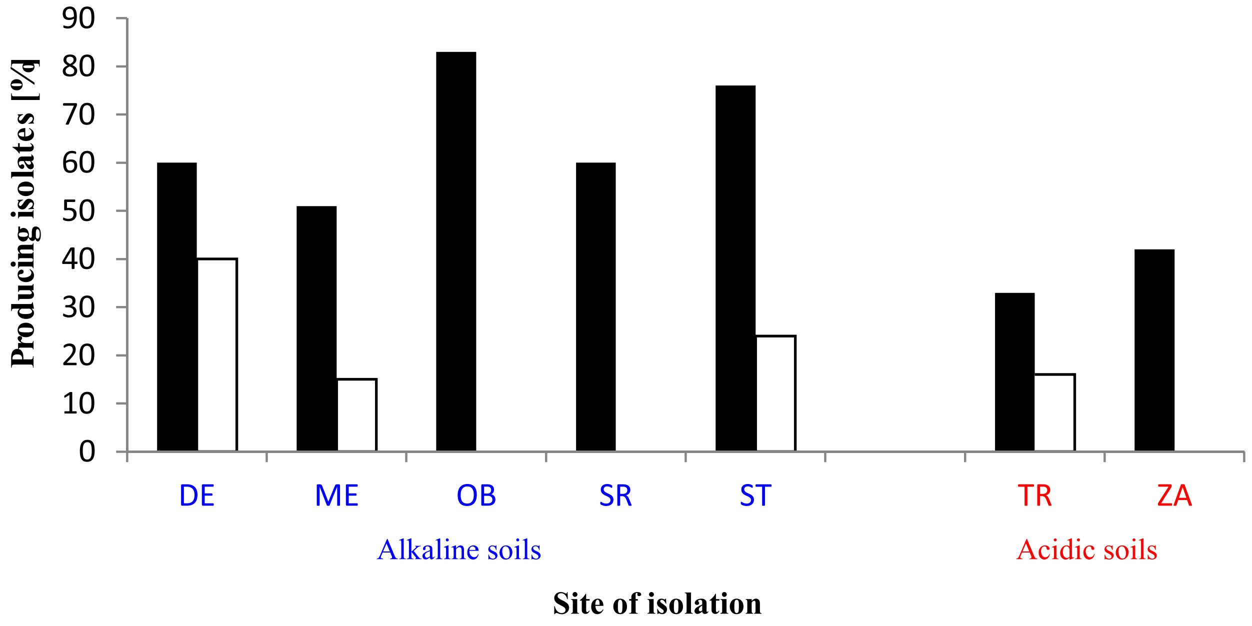

Figure S5. Low molecular weight compounds detected by TLC analysis.

The columns indicate a percentage of strains isolated from alkaline (blue) and acidic (red) soils, in which a compound of the specified R_f value was detected.

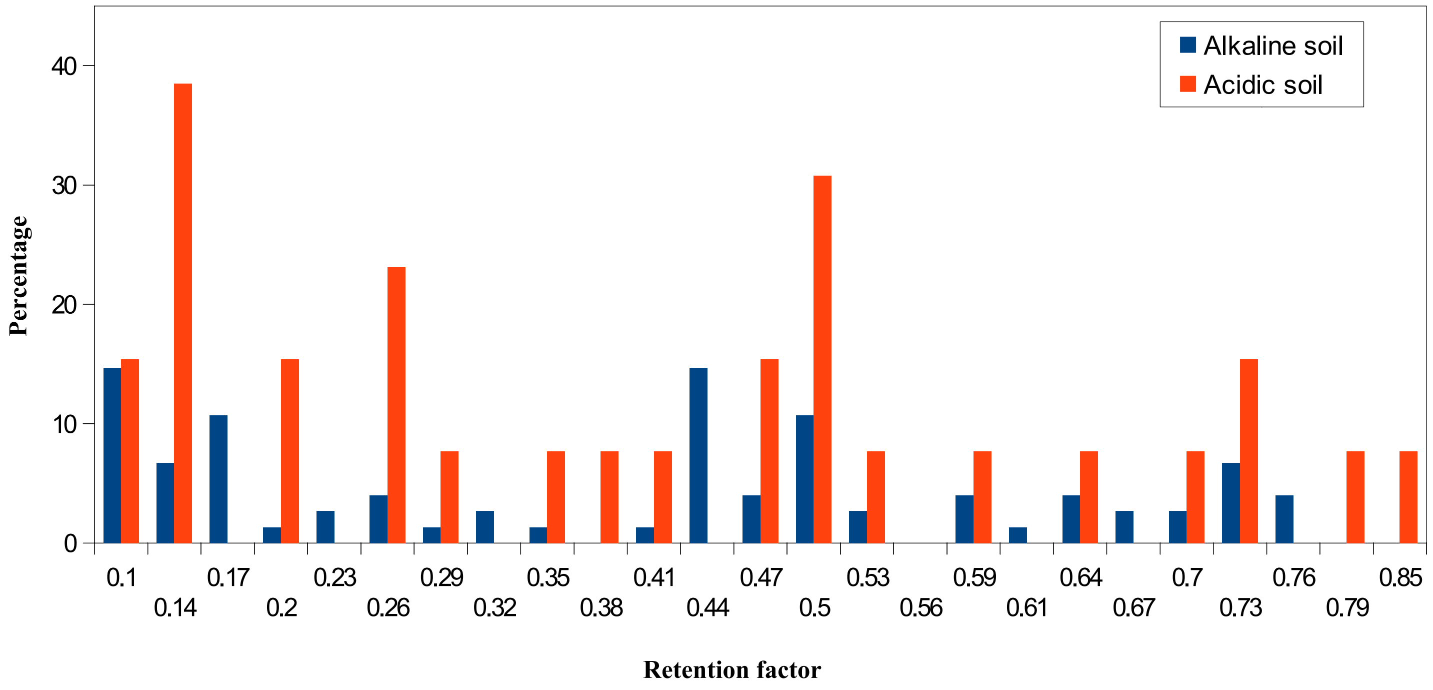

Supplement: Additional file 1: — Figure S1. In silico analysis of primer coverage with sequence sets retrieved from 306 genomes of Actinobacteria in JGI_IMG database. Figure S2. Phylogenetic analysis of actinobacterial methyltransferase sequences related to Erm. Figure S3. Phylogenetic analysis of actinobacterial ABC transporter sequences. Figure S4. The proportion of strains expressing antibiotic activity. Figure S5. Low molecular weight compounds detected by TLC analysis. [file 12866_2015_416_MOESM1_ESM.pdf]
